# ANGPTL8 promotes adipogenic differentiation of mesenchymal stem cells: potential role in ectopic lipid deposition

**DOI:** 10.3389/fendo.2022.927763

**Published:** 2022-08-11

**Authors:** Jian Tang, Shinan Ma, Yujiu Gao, Fan Zeng, Ying Feng, Chong Guo, Lin Hu, Lingling Yang, Yanghui Chen, Qiufang Zhang, Yahong Yuan, Xingrong Guo

**Affiliations:** ^1^ Department of Neurosurgery, Hubei Key Laboratory of Embryonic Stem Cell Research, Taihe Hospital, Hubei University of Medicine, Shiyan, China; ^2^ Central Laboratory, Xiangyang Central Hospital, Affiliated Hospital of Hubei University of Arts and Science, Xiangyang, China; ^3^ Department of Geriatrics & General Medicine, Affiliated Taihe Hospital of Hubei University of Medicine, Shiyan, China; ^4^ Hubei Clinical Research Center for Umbilical Cord Blood Hematopoietic Stem Cells, Taihe Hospital, Hubei University of Medicine, Shiyan, Hubei, China

**Keywords:** ectopic lipid accumulation, Wnt/ß-Catenin pathway, ANGPTL8, mesenchymal stem cells, adipogenic differentiation

## Abstract

**Background:**

Ectopic lipid deposition plays a promoting role in many chronic metabolic diseases. Abnormal adipogenic differentiation of mesenchymal stem cells (MSCs) is an important cause of lipid deposition in organs. Studies have shown that serum angiopoietin-like protein 8 (ANGPTL8) levels are increased in patients with many chronic metabolic diseases (such as type 2 diabetes, obesity, and hepatic steatosis), while the role of ANGPTL8 in ectopic lipid accumulation has not been reported.

**Methods:**

We used the Gene Expression Omnibus (GEO) database to analyze the expression of ANGPTL8 in subcutaneous adipose tissue of obese patients and qPCR to analyze the expression of ANGPTL8 in the liver of high-fat diet (HFD)-induced obese mice. To explore the potential roles of ANGPTL8 in the progression of ectopic lipid deposition, ANGPTL8 knockout (KO) mice were constructed, and obesity models were induced by diet and ovariectomy (OVX). We analyzed lipid deposition (TG) in the liver, kidney, and heart tissues of different groups of mice by Oil Red O, Sudan black B staining, and the single reagent GPO-PAP method. We isolated and characterized MSCs to analyze the regulatory effect of ANGPTL8 on Wnt/β-Catenin, a key pathway in adipogenic differentiation. Finally, we used the pathway activator LiCl and a GSK3β inhibitor (i.e., CHIR99021) to analyze the regulatory mechanism of this pathway by ANGPTL8.

**Results:**

ANGPTL8 is highly expressed in the subcutaneous adipose tissue of obese patients and the liver of HFD-induced obese mice. Both normal chow diet (NCD)- and HFD-treated ANGPTL8 KO male mice gained significantly less weight than wild-type (WT) male mice and reduced ectopic lipid deposition in organs. However, the female mice of ANGPTL8 KO, especially the HFD group, did not show differences in body weight or ectopic lipid deposition because HFD could induce estrogen overexpression and then downregulate ANGPTL8 expression, thereby counteracting the reduction in HFD-induced ectopic lipid deposition by ANGPTL8 deletion, and this result was also further proven by the OVX model. Mechanistic studies demonstrated that ANGPTL8 could promote the differentiation of MSCs into adipocytes by inhibiting the Wnt/β-Catenin pathway and upregulating PPARγ and c/EBPα mRNA expression.

**Conclusions:**

ANGPTL8 promotes the differentiation of MSCs into adipocytes, suggesting that ANGPTL8 may be a new target for the prevention and treatment of ectopic lipid deposition in males.

## Introduction

According to statistical data from the World Health Organization (WHO), the prevalence of obesity (body mass index (BMI)>30 kg/m2) in adults has been increasing worldwide over the past few decades (1). Obesity arises from chronic energy imbalance, where energy intake exceeds energy expenditure, resulting in ectopic accumulation of harmful lipids in multiple tissues (2). Ectopic lipid deposition is associated with the development of insulin resistance (3), type 2 diabetes (4), and nonalcoholic fatty liver disease (5). Nonetheless, the mechanism of ectopic lipid deposition is still not fully understood, and further exploration is needed.

Mesenchymal stem cells (MSCs) are pluripotent stem cells with self-renewal and multidirectional differentiation potential that can differentiate into a variety of stromal tissues, such as bone, cartilage, and fat. MSCs are widely distributed in the body, existing in bone marrow, muscle, adipose tissue, liver, pancreas, and other organs, so the differentiation function of MSCs has a certain impact on the repair and structural composition of the tissues and organs (6). Studies have shown that the differentiation tendency of MSCs is closely related to the homeostasis of the internal environment. When human body fat exceeds the normal threshold and reaches the level of obesity, the body will have relatively abnormal homeostasis, and the differentiation trend of MSCs will also undergo corresponding abnormal changes. The MSCs of obese mice were found to be enhanced *in vitro* to differentiate into adipocytes induced by high fat (7). Clinical studies have also found that MSCs in the adipose tissue of obese patients are more likely to differentiate into adipose cells to increase the number of adipose cells and meet the body’s demand for excess energy storage (8, 9). Therefore, the abnormal differentiation of MSCs to adipocytes in the body is one of the key causes of obesity. The current study shows that the increase in the number of adipocytes promotes the occurrence of obesity due to the role of a type of obesogen (10), but it is not completely clear which obesogens induce MSCs to be more likely to differentiate into adipocytes.

Angiopoietin-like protein 8 (ANGPTL8), a recently identified member of the angiopoietin-like protein family, is a secretory protein highly expressed in liver and adipose tissue. Studies have shown that members 1-7 of the ANGPTL family have similar structures but different functions. ANGPTL8 lacks a common structure with ANGPTL1-7 but has homology with the nitrogenterminal domain of ANGPTL3, which makes ANGPTL8 unique in the angiopoietin-like protein family (11). ANGPTL8 was shown to have an important role in lipid metabolism. In obese mice induced by high-fat feeding, the content of ANGPTL8 in the liver and serum increased significantly, and the weight and serum triglyceride concentration of ANGPTL8 knockout mice decreased significantly (12). In addition, the ANGPTL8 level in the serum of obese patients also increased significantly (13), and ANGPTL8 was shown by Xue et al. (14) to promote the differentiation of adipocytes. Based on these previous studies, we speculate that ANGPTL8 may be a potential obesogen. However, it is unclear whether ANGPTL8 promotes ectopic lipid deposition by regulating the adipogenic differentiation of MSCs.

In this study, the effect of ANGPTL8 on mouse body weight and organ fat deposition induced by NCD or HFD was measured in ANGPTL8 KO mice. Furthermore, our study confirmed that ANGPTL8 could promote the adipogenic differentiation of MSCs by regulating Wnt/β-catenin signaling *in vitro*. And ANGPTL8 could lead to multiorgan ectopic lipid deposition. This study elucidates a novel mechanism of action of ANGPTL8 in adipogenesis and provides a potential molecular basis for the prevention and treatment of ectopic lipid deposition.

## Materials and methods

### Animals

ANGPTL8−/− (ANGPTL8 KO) mice were generated in C57BL/6J mice by the clustered regularly interspaced short palindromic repeats (CRISPR)/Cas9 system using a previously described method in the Laboratory Animal Center of Sun Yat-sen University (15). All the F0 and F1 generation animals were obtained at the Laboratory Animal Center of Sun Yat-sen University, and then the F1 generation mice identified as ANGPTL8 knockout (KO) heterozygotes were transferred to the specific pathogen-free (SPF) Animal Experimental Center of the Hubei University of Medicine for further experimental study.

Mice were housed (three to five per cage) in a controlled environment (12-h dark/12-h light daily cycle, 60–70% humidity, 23 ± 1°C). Six- to eight-week-old male C57BL/6J wild-type or ANGPTL8 KO mice (all the mice were strictly littermates and cohoused, 5-10 mice per group) were fed a high-fat diet (HFD –60.9% fat, 18.3% protein and 21.8% carbohydrate; D12492; Research Diets) for 20 months, with a normal chow diet (NCD; 78% carbohydrate, 4% fat, and 18% protein; D12450J; Research Diets) as a control. We investigated the impact of ANGPTL8 on ectopic fat metabolism using *in vivo* experiments with ANGPTL8 KO mice fed an HFD and NCD for up to 10 or 20 months. Five to eight mice per group of 8-week-old female mice were subjected to OVX. The sham operation Group, in which the ovaries were exteriorized and then replaced into the abdominal cavity as a control.

The mice were deprived of food for 6 h before sacrifice and anesthetized with sodium pentobarbital (50 mg/kg, administered intraperitoneally). Blood samples were rapidly collected *via* cardiac puncture blood collection and the mice were immediately sacrificed by cervical dislocation. Inguinal, retroperitoneal, and mesenteric fat pads, liver, kidney, and heart were dissected and weighed.

### GEO data analysis

Subcutaneous fat in obese patients and normal male and female liver RNA-Seq datasets were downloaded from the GEO database (GEO: GSE156906 and GEO: GSE55668) (https://www.ncbi.nlm.nih.gov/geo/). The raw data were downloaded as MINiML files. The extracted data were normalized by log2 transformation. The microarray data were normalized by the normalized quantiles function of the preprocessCore package in R software (version 3.4.1). Probes were converted to gene symbols according to the annotation information of the normalized data in the platform (16).

### Isolation and preparation of hADSCs and hUMSCs

Human adipose and umbilical cord samples were taken from patients with full-term caesarian sections, with their consent and approved by The TaiHe Hospital Affiliated to the Hubei University of Medicine. All procedures were conducted by the guidelines of the Medical Ethics Committee of the Health Bureau. The procedure for the isolation of human adipose-derived mesenchymal stem cells (hADSCs) was performed according to a previous study (17). First, fresh adipose tissue was washed with phosphate-buffered saline (PBS) (0.15 M, pH 7.4) and penicillin/streptomycin (5%) (New Cell & Molecular Biotech Co., Ltd., China), and collagen digestion was performed for 1 hour. Collagenase type IV (Thermo Fisher Scientific, USA) dissolved in PBS (1 mg/mL) was used following extensive shaking at 37°C in a humid atmosphere containing 5% CO^2^. Then, the collagenase solution was neutralized by adding the same volume of complete growth medium containing Dulbecco’s modified Eagle’s medium (DMEM)/F12 (Cytiva, USA) supplemented with 10% fetal bovine serum (FBS) (Thermo Fisher Scientific, USA), 100 µg/mL penicillin/streptomycin, 1 g/mL amphotericin B (New Cell & Molecular Biotech Co., Ltd., China), 5 ng/mL epidermal growth factor (Invitrogen; Thermo Fisher Scientific, Inc., USA), and 5 ng/mL basic fibroblast growth factor (Invitrogen; Thermo Fisher Scientific, Inc., USA). In the following steps, the blood cells were separated with red blood cell (RBC) lysis buffer (pH=7.3) and centrifuged. The attached cells were resuspended in a complete growth medium and transferred to a T-25 flask. Human umbilical cord mesenchymal stem cells (hUMSCs) were isolated from the gelatinous tissue around the vein and the artery according to our previous study (18). The morphological shapes and specified cluster differentiation (CD) markers were studied to authenticate the specific hADSCs or hUMSCs.

### Flow cytometry analysis

Flow cytometry analysis was used to determine the hADSC- and hUCMSC-specific markers. Subconfluent hADSCs and hUC-MSCs were detached with 0.25% trypsin and washed with PBS. A total of 1-5×10^5^ cells were resuspended in 200 mL of PBS for each reaction. Cells were incubated on ice for 30 min with the following mouse antihuman antibodies, which were conjugated with fluorescein isothiocyanate (FITC) or phycoerythrin (PE): CD90-FITC, CD73-FITC, and CD105-FITC and negative ones including CD45-PE, CD34-PE, CD31-FITC, HLA-DR-FITC, CD14-PE and CD19-PE (Biolegend, USA). Mouse immunoglobulin (Ig)G1-PE and mouse IgG1-FITC were used as isotype controls. Cells were analyzed using flow cytometry (FACScan, BD Biosciences, San Jose, CA, USA).

### *In vitro* differentiation

Cultured hADSCs and hUMSCs (5>passage number>3, approximately 1×10^6^ cells) were induced to differentiate into adipocytes using a differentiation medium. The medium contained 1 µM dexamethasone, 10 µg/mL insulin, 100 µM indomethacin, and 0.5 mM 3-isobutyl-1-methylxanthine (IBMX) (all from Cyagen Biotechnology Co., Ltd., China). The prepared cells were also treated with 400 ng/mL of human recombinant protein ANGPTL8 (rANGPTL8, Novoprotein, China) from the beginning of differentiation. hADSCs and hUMSCs were differentiated with an induction medium for 24 days. Oil Red O lipid staining was performed to illustrate lipid droplets. Briefly, Oil Red O (Sigma, USA) working solution (0.3% Oil Red O dissolved in 0.18% isopropanol) was added to the fixed cells (in 10% formalin) and incubated for 30 min at room temperature (RT). The cells were washed and examined and photographed using an inverted light microscope (Olympus IX53, Japan). For the quantification of Oil Red O staining, the stain was extracted in 0.4 mL of 100% isopropanol, and 0.2 mL was used to measure Oil Red O staining in a 96-well plate at OD540 nm.

### Immunofluorescence

Cultured hADSCs were fixed with 4% paraformaldehyde for 15 min at RT, permeabilized with 0.1% Triton X-100 for 5 min at RT, washed three times with PBS, and blocked with 5% bovine serum albumin (BSA) in TBST for 30 min. hADSCs were incubated with rabbit polyclonal antibody against β-catenin (1:200) overnight at 4°C and then washed 3 times with PBS. hADSCs were incubated with FITC-conjugated goat anti-mouse IgG (H+L) (1:200, Beyotime Biotechnology, China) for 2 h at RT and washed 3 times with PBS, and the nuclei were stained with diamidino-2-phenylindole (DAPI) (Beyotime Biotechnology, China). The intensity of immunofluorescence was analyzed by Fiji ImageJ software.

### Quantitative real-time polymerase chain reaction (PCR) analysis

Total RNA was extracted using TRIzol total RNA isolation reagent (LABLEAD, China), and cDNA was synthesized using All in One First-Strand Synthesis MaterMix (LABLEAD, China). Quantitative real-time PCR (qRT–PCR) was carried out using QuantiNova™ SYBR^®^ Green PCR Master Mix (QIAGEN, Germany) in a real-time PCR instrument (Bio-Rad Laboratories, Inc., USA). cDNA samples were diluted at 1:10 for all analyses, which were performed in quadruplicate. Expression values were obtained using the ΔΔCt method and normalized to β-actin expression; average values are shown as the mean ± standard deviation (s.d.). The primer sequences of the genes were designed according to the sequence information from the GenBank database (**Table 1**).

**Table 1:** Primer sequences are used for RT-PCR.

Gene		Primer Sequences (5’-3’)
mouse ANGPTL8	Forward Reverse	GACACTGTACGGAGACTAC AGGTGGCTCTGCTTATCA
mouse β-actin	Forward Reverse	ACTGAGCTGCGTTTTACAC GATGTTTGCTCCAACCAACT
mouse C/EBPα	Forward Reverse	GATGAGCAGTCACCTCCAGAG GCCAGGAACTCGTCGTTGA
mouse PPARγ	Forward Reverse	TCTGCTCCACACTATGAAGACA CCACAGACTCGGCACTCAA
human C/EBPα	Forward Reverse	ACAAGAACAGCAACGAGTACC CGGTCATTGTCACTGGTCAG
human PPARγ	Forward Reverse	TCCACATTACGAAGACATTCCA CTCCACAGACACGACATTCAA
Human β-actin	Forward Reverse	GCACCACACCTTCTACAATGAG GATAGCACAGCCTGGATAGCA

### Western blot analysis

Western blot analysis was performed using standard procedures. Cells were lysed in radioimmunoprecipitation assay (RIPA) lysis buffer (Beyotime Biotechnology, China). The protein was prepared and quantified by Bradford analysis (Beyotime, China). The same amounts of protein were extracted by 10% and transferred onto a polyvinylidene fluoride (PVDF) membrane (Millipore, Germany). Membranes were blocked with 5% milk in Tris-buffered saline with Tween 20 (TBST) for 1 hour and incubated with primary antibody for 2 hours at RT or overnight at 4°C. The membranes were washed 3 times with PBST and then incubated with species-specific secondary antibodies for 1.5 hours at RT. Next, the membranes were washed 3 times with TBST, once with tris-buffered saline (TBS), and then imaged using the Bio-Rad chemiluminescence imaging system (Bio-Rad Laboratories, Inc., USA). All image quantifications were performed using Fiji ImageJ software.

The following primary antibodies were used: rabbit polyclonal antibody against β-catenin (Proteintech Group, China), rabbit polyclonal antibody against GSK3β (Beyotime, China), and mouse polyclonal antibody against β-actin (Beyotime, China). The following secondary antibodies were used: goat anti-mouse IgG/AP (Beyotime, China) and goat anti-rabbit IgG/HRP (Beyotime, USA).

### Oil red O and Sudan black B staining to analyze tissue lipid deposition

Oil Red O staining was performed on frozen sections. Briefly, frozen sections at 5-μm thickness were fixed with 4% paraformaldehyde for 10 min, washed with PBS, soaked in Oil Red O working solution for 30 min, washed and observed, and photographed under an inverted light microscope. Sudan Black B staining was performed on frozen sections. Briefly, frozen sections at 5-μm thickness were fixed with 4% paraformaldehyde for 10 min, washed with PBS, and then soaked in Sudan black B working solution (Sigma, China) for 30 min. Hematoxylin counterstaining was performed to visualize nuclei, which were then washed and observed, and photographed under an inverted light microscope.

### Primary mouse hepatocyte isolation

The procedure was performed according to a previous study (19, 20). First, the mouse livers were perfused with collagenase (type IV, 100 CDU/mL, Sigma, USA) as liver perfusion reagents, and the liver was excised rapidly and placed into cold Hanks’ balanced salt solution (HBSS). The primary hepatic cells were released and filtered by a sterile 70-μm filter (Beyotime; FSTR070) and then centrifuged for 5 minutes at 4°C at 50 g. The hepatocytes were in the pellet. The hepatocytes were resuspended in 20 mL of DMEM (HyClone, Germany) containing 10% FBS for primary hepatocyte culture.

### Measurement of intracellular and hepatic TG contents

For intracellular TG content, the cells were fixed, and Oil Red O was extracted. Then, the extracted cells were diluted in 100% isopropanol and measured at 540 nm using a microplate reader.

For hepatic triglyceride content, lipids were measured using an EnzyChrom™ Triglyceride Assay Kit (BioAssay Systems) and normalized to wet tissue weight.

### Statistical analysis

Statistical analysis was performed by using GraphPad Prism software (GraphPad Prism 8). Statistical significance was calculated with a one-way analysis of variance (ANOVA) corrected for multiple comparisons. Fold change regulation, comparing the treatment group versus the control group, was evaluated using Student’s t-test. Data are presented as the mean ± standard error of the mean (SEM). A P value < 0.05 was considered statistically significant.

## Results

### ANGPTL8 correlated positively with the incidence of obesity

**Figure 1:**
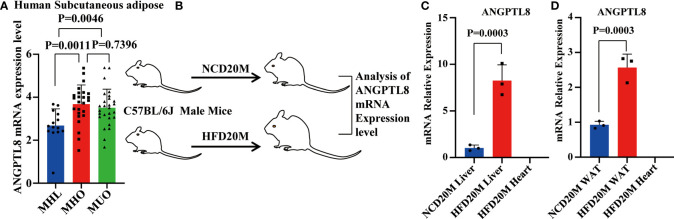
The mRNA expression of ANGPTL8 positively correlates with obesity. **(A)** Differential expression of ANGPTL8 mRNA levels in subcutaneous adipose tissue transcriptomic data from the GEO database of obese populations (n ≥ 14). **(B)** Schematic of the experimental design and sample processing. **(C)** Analysis of ANGPTL8 mRNA expression in obese male mouse livers fed an HFD for 20 months by RT–qPCR (n = 3). HFD20 M mouse hearts were used as a negative control. **(D)** Analysis of ANGPTL8 mRNA expression in WAT from obese male mice fed an HFD for 20 months by RT–qPCR (n = 3). HFD20 M mouse hearts were used as a negative control.

To evaluate whether ANGPTL8 expression is associated with obesity, we first analyzed publicly available transcriptomic data of subcutaneous adipose tissue from a cohort of obese individuals extracted from the GEO database. These data (21) include subcutaneous adipose tissue transcription data of metabolically healthy lean people with normal triglyceride content in the liver (MHL), and metabolically healthy obese people with normal triglyceride content in the liver (MHO), and metabolically unhealthy obese people with nonalcoholic fatty liver (MUO). As shown in **Figure 1A**, we found that the expression of ANGPTL8 was significantly upregulated in subcutaneous adipose tissue of MHO and MUO compared to MHL.

Meanwhile, we detected the expression levels of ANGPTL8 in mice with HFD-induced obesity. Previous studies demonstrated that ANGPTL8 is expressed mainly in the liver and adipose tissue in mice, so we tested ANGPTL8 expression in liver and white adipose tissue (WAT) of HFD 20 M mice by RT–qPCR (**Figure 1B**). Our results showed that the levels of ANGPTL8 were significantly higher in the 20-month HFD group than in NCD-treated mice, with the most significantly elevated expression in the liver and WAT (**Figures 1C** **, D**). All results indicate that upregulation of ANGPTL8 expression is associated with the incidence of obesity.

### ANGPTL8 deficiency reduces diet-induced ectopic lipid deposition in male mice

**Figure 2:**
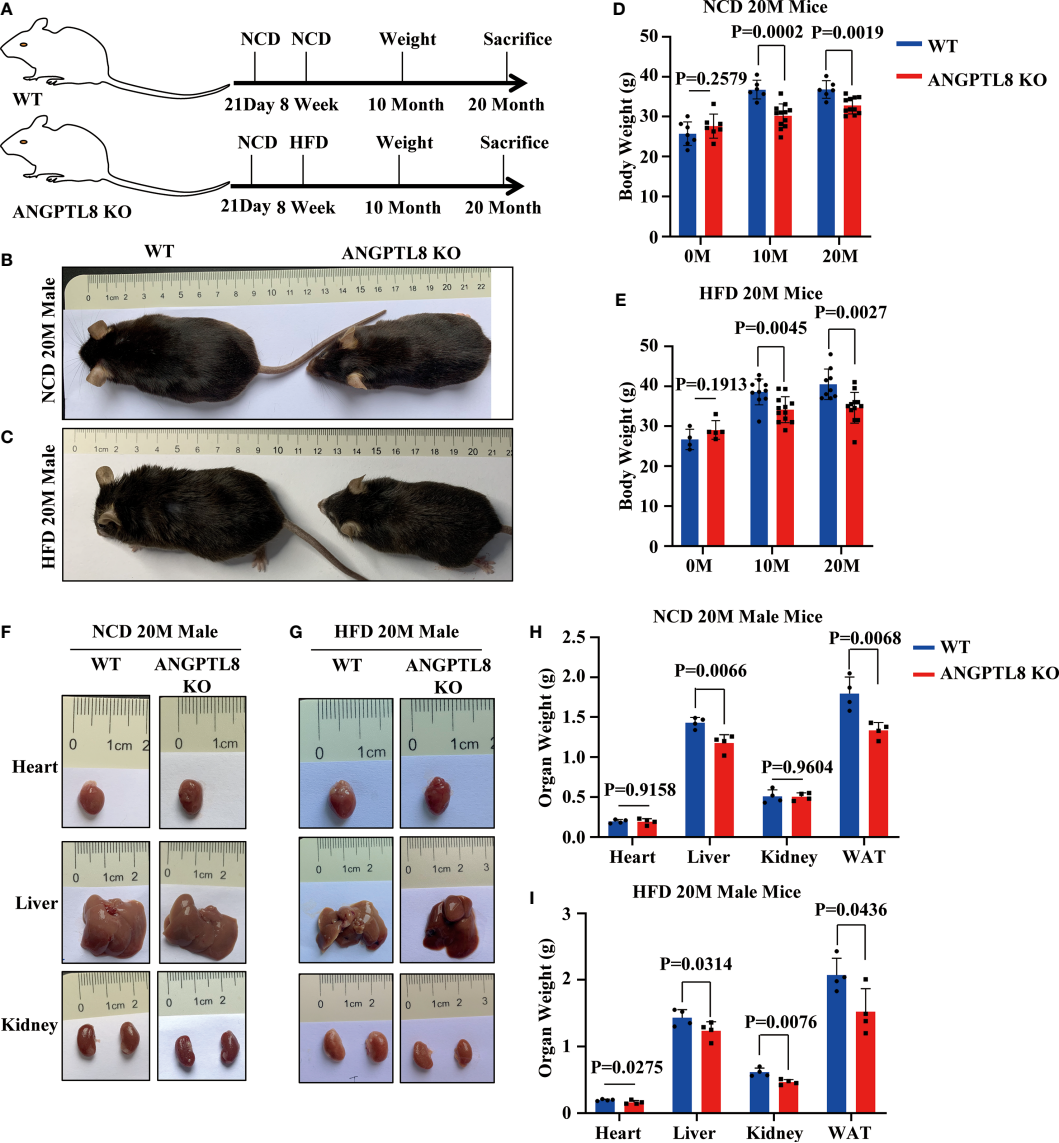
ANGPTL8 knockout suppresses diet-induced obesity in male mice. **(A)** Schematic of the experimental design. **(B)** The overall appearance of WT and ANGPTL8 KO male mice fed an NCD for 20 months. **(C)** The overall appearance of WT and ANGPTL8 KO male mice fed an HFD for 20 months. **(D)** Body weight change of WT and ANGPTL8 KO male mice at 10 and 20 months after NCD feeding (n ≥ 6). **(E)** Body weight change of WT and ANGPTL8 KO male mice at 10 and 20 months after HFD feeding (HFD started at 8 weeks old, n ≥ 4). **(F)** Macroscopic heart, liver, and kidney appearance of ANGPTL8 KO and WT male mice fed an NCD for 20 months. **(G)** Macroscopic heart, liver, and kidney appearance of ANGPTL8 KO and WT male mice fed an HFD for 20 months. **(H)** The weights of the heart, liver, kidney, and white adipose tissue of ANGPTL8 KO male mice and WT male mice fed an NCD for 20 months (n = 4). **(I)** The weights of the heart, liver, kidney and white adipose tissue of ANGPTL8 KO male mice and WT male mice fed an HFD for 20 months (n = 4).

To explore the potential roles of ANGPTL8 in the progression of diet-induced obesity, we first constructed ANGPTL8 knockout mice (KO) (**Supplementary Figures 1A–C**) and investigated the impact of ANGPTL8 on body fat synthesis to induce lipid deposition in multiple organs of ANGPTL8 KO and WT mice after 10 and 20 months (10 M and 20 M) of NCD or HFD. The results showed that ANGPTL8 KO inhibited body weight in both NCD- and HFD-treated male mice after induction for 10 and 20 M (**Figures 2A** **–E**), and only homozygotes but not heterozygotes showed significant differences compared with WT (**Supplementary Figure 2A**). At the same time, we analyzed the organ weight of mice and found that ANGPTL8 KO significantly inhibited the weight of liver and white adipose tissue, and there was no significant difference between the heart and kidney in NCD-treated 20 M male mice (**Figures 2F** **, H**). However, ANGPTL8 deficiency significantly inhibited the weight of the heart, liver, kidney, and white adipose tissue in HFD-fed 20 M male mice (**Figures 2G** **, I**), and the decrease in WAT and liver accounted for the change in body weight (**Supplementary Figures 2B, C**).

**Figure 3:**
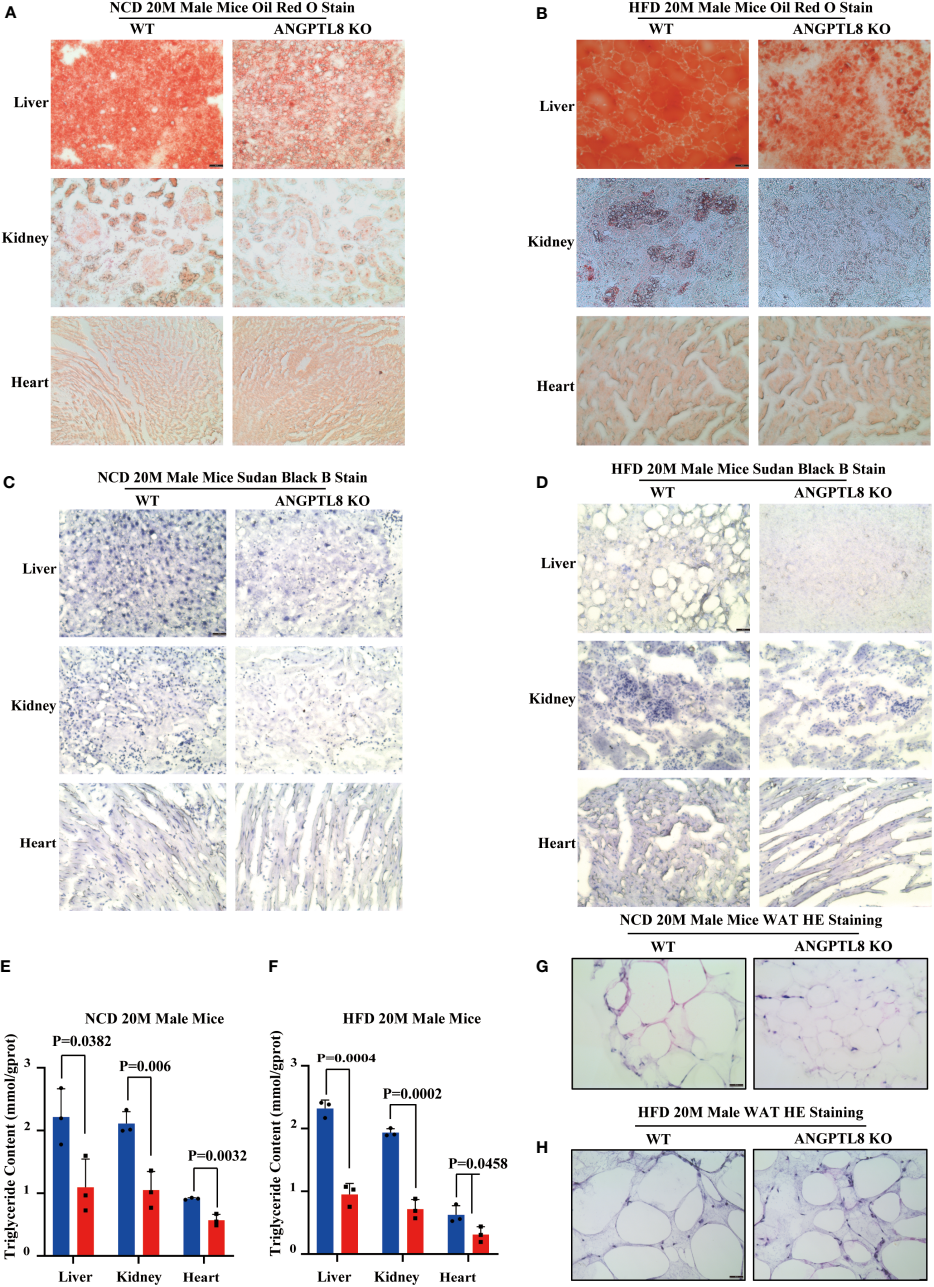
ANGPTL8 deletion suppresses diet-induced ectopic lipid deposition in the liver, kidney, heart, and WAT of male mice. **(A)** Micrographs showing Oil Red O staining of the frozen liver, kidney, and heart sections from male WT and ANGPTL8 KO mice after NCD feeding for 20 months (red = Oil Red O, scale bar = 50 μm, n = 5). **(B)** Micrographs showing Oil Red O staining of frozen liver, kidney, and heart sections from male WT and ANGPTL8 KO mice after HFD consumption for 20 months (red = Oil Red O, scale bar = 50 μm, n = 5). **(C)** Micrographs showing Sudan Black B staining of frozen liver, kidney, and heart sections from male WT and ANGPTL8 KO mice after NCD feeding for 20 months (scale bar = 50 μm, n = 5). **(D)** Micrographs showing Sudan Black B staining on frozen liver, kidney, and heart sections in male WT and ANGPTL8 KO mice after HFD consumption for 20 months (scale bar = 50 μm, n = 5). **(E)** Quantitative analysis of TG in the liver, kidney, and heart of ANGPTL8-KO and WT male mice treated with NCD for 20 months. **(F)** Quantitative analysis of TG in the liver, kidney, and heart of ANGPTL8-KO and WT male mice fed an HFD for 20 months. **(G)** Representative HE staining of white adipose tissue of male WT and ANGPTL8 KO mice after NCD for 20 months (scale bar = 50 μm, n = 5). **(H)** Representative HE staining of white adipose tissue of male WT and ANGPTL8 KO mice after HFD consumption for 20 months (scale bar 50 = μm, n = 5).

To further elucidate the contribution of ANGPTL8 to diet-induced differences in organ weights, we first analyzed lipid deposition (TG) in liver, kidney, and heart tissues of NCD- and HFD-fed 20 M male mice by Oil Red O staining. As shown in **Figures 3A** **, B**, ANGPTL8 KO significantly inhibited lipid droplet deposition in the liver and kidney in NCD- and HFD-fed male mice and the change in the heart was only in HFD-fed mice but not in NCD-fed male mice. We next analyzed the differential effects of ANGPTL8 on lipid accumulation (including phospholipids, sphingolipids, glycolipids, steroids, etc.) in the liver, kidney, and heart of mice by Sudan black B staining and found that ANGPTL8 KO significantly inhibited the accumulation of lipids in both NCD-fed and HFD-fed 20 M male mouse organs (**Figures 3C** **, D**). The results of quantitative analysis of TG in liver, kidney, and heart tissue showed that the extent of lipid deposition in these tissues was reduced in the ANGPTL8 KO group compared to the WT mice (**Figures 3E** **, F**). Hematoxylin and eosin (HE) staining also showed that the volume of adipocytes in the white adipose tissue (WAT) of ANGPTL8 KO male mice was significantly smaller than the volume of WT adipocytes (**Figures 3G** **, H**). All the above results suggest that ANGPTL8 KO contributes to obesity by reducing the deposition of various classes of lipids in the tissue organs of male mice.

### ANGPTL8 did not affect diet-induced ectopic lipid accumulation in female mice

**Figure 4:**
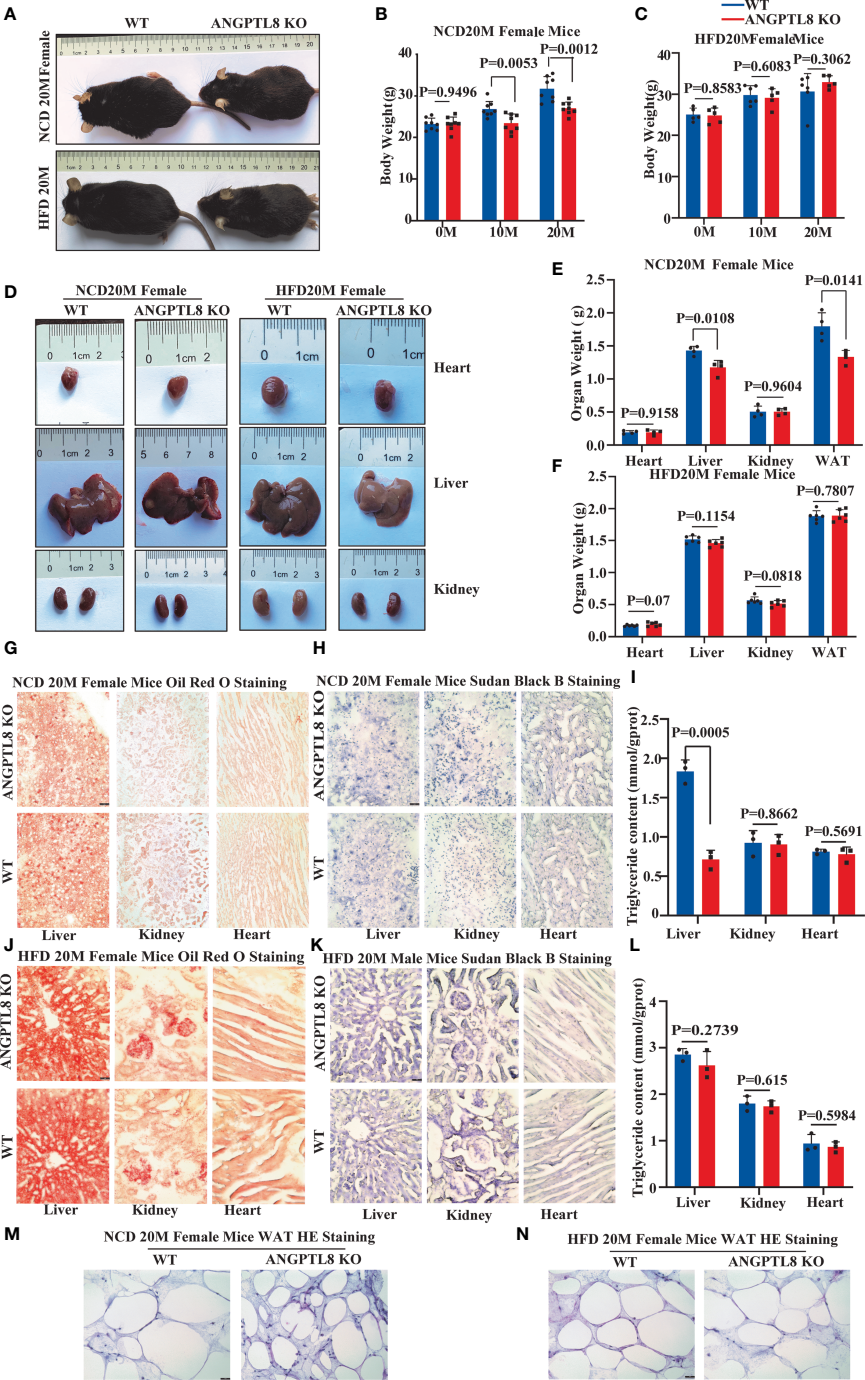
The effect of ANGPTL8 on diet-induced obesity and lipid deposition in female mice. **(A)** Gross appearance of ANGPTL8 KO and WT female mice fed an NCD or HFD for 20 months. **(B, C)** Body weight change of WT and ANGPTL8 KO female mice at 10 and 20 months after NCD **(B)** (n = 8) and HFD feeding (n ≥ 5). **(D)** Macroscopic heart, liver, and kidney appearance of ANGPTL8 KO and WT female mice fed an NCD or HFD for 20 months. **(E)** and **(F)** The weights of the heart, liver, kidney, and white adipose tissue of ANGPTL8 KO and WT female mice fed an NCD (n = 4) and HFD for 20 months (n = 6). **(G)** Micrographs showing Oil Red O staining of the frozen liver, kidney, and heart sections in female WT and ANGPTL8 KO mice after NCD feeding for 20 months (red = Oil Red O. **(H)** Micrographs showing Sudan Black B staining of the frozen liver, kidney, and heart sections from female WT and ANGPTL8 KO mice after NCD feeding for 20 months. **(I)** Quantitative analysis of TG in the liver, kidney, and heart of ANGPTL8-KO and WT female mice treated with NCD for 20 months. **(J)** Micrographs showing Oil Red O staining of frozen liver, kidney, and heart sections from female WT and ANGPTL8 KO mice after HFD consumption for 20 months. **(K)** Micrographs showing Sudan Black B staining on frozen liver, kidney, and heart sections in female WT and ANGPTL8 KO mice after HFD consumption for 20 months. **(L)** Representative HE staining of white adipose tissue of female WT and ANGPTL8 KO mice after HFD consumption for 20 months. **(L)** Quantitative analysis of TG in the liver, kidney, and heart of ANGPTL8-KO and WT female mice fed an HFD for 20 months. **(M, N)** Representative HE staining of white adipose tissue of female WT and ANGPTL8 KO mice after NCD and HFD feeding for 20 months. (red = Oil Red O. (scale bar = 50 μm, n = 5).

**Figure 5:**
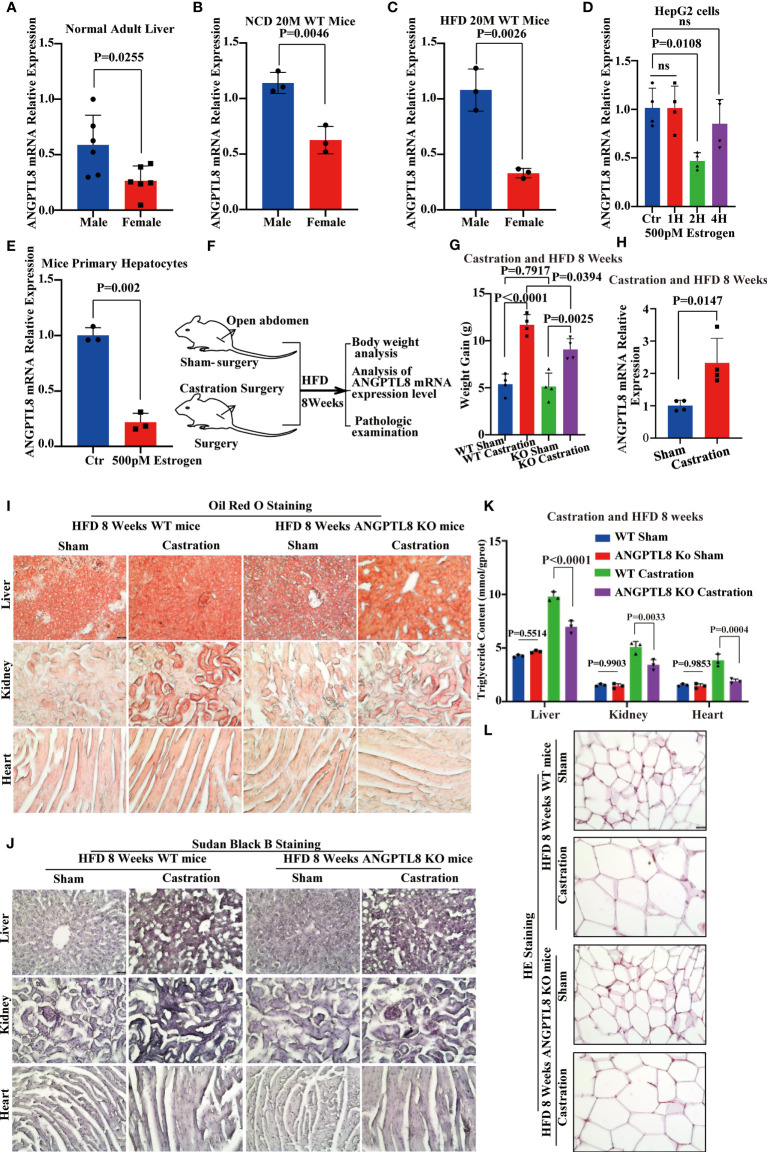
Estrogen protects against high-fat diet-induced ectopic lipid deposition by inhibiting ANGPTL8 expression. **(A)** Differential expression of ANGPTL8 mRNA in normal male and female livers (n = 6). **(B)** Differential expression of ANGPTL8 mRNA in NCD 20 M male and female mouse livers (n = 3). **(C)** Differential expression of ANGPTL8 mRNA in HFD 20 M male and female mouse livers (n = 3). **(D)** Analysis of the relative mRNA expression levels of ANGPTL8 in HepG2 cells after treatment with 500 pM estrogen for 0, 1, 2, and 4 hours (n = 4). **(E)** Analysis of the relative mRNA expression levels of ANGPTL8 in mouse primary hepatocytes after treatment with 500 pM estrogen for 2 hours (n = 3). **(F)** Schematic of the experimental design. **(G)** Body weight change of WT female mice fed an HFD for 8 weeks after castration surgery (n = 4). **(H)** Differential expression of ANGPTL8 mRNA in HFD 8-week-old female mouse livers after castration surgery (n = 3). **(I)** Micrographs showing Oil Red O staining on the frozen liver, kidney, and heart sections in female WT mice after castration surgery and HFD 8 weeks (scale bar = 50 μm, n = 4). **(J)** Micrographs showing Sudan Black B staining on the frozen liver, kidney, and heart sections in female WT mice after castration surgery and HFD 8 weeks (scale bar = 50 μm, n = 4). **(K)** Quantitative analysis of TG in the liver, kidney, and heart of female WT mice after castration surgery and HFD 8 weeks (scale bar = 50 mm, n = 4). **(L)**. Representative HE staining of white adipose tissue of female WT mice after castration surgery and HFD 8 weeks (scale bar = 50 μm, n = 4).

We found an interesting phenomenon in which the effect of ANGPTL8 on diet-induced obesity was not obvious in female mice. After 10 and 20 months of NCD feeding, ANGPTL8 KO reduced weight, especially liver weight, WAT weight, and lipid deposition (**Figure 4N**). However, after 10 and 20 months of HFD feeding, ANGPTL8 deficiency had no significant effect on body weight, organ weight, or lipid deposition (**Figures 4A** **–N**). We hypothesized that the different roles of ANGPTL8 in diet-induced ectopic lipid deposition in male and female mice were due to the inhibition of estrogen on ANGPTL8 expression. Our analysis of the GEO database revealed that ANGPTL8 expression is higher in the liver of males than in females in the normal population (**Figure 5A**). Then, we examined the expression of ANGPTL8 in the livers of male and female mice fed an NCD or HFD for 20 months, as shown in **Figures 5B** **, C**. ANGPTL8 expression was lower in the livers of female mice fed an NCD or HFD than in male mice. In particular, the decrease was most pronounced in HFD-fed females. The addition of estrogen to the growth media of human HepG2 cells and mouse liver hepatocyte cells for 2 hours significantly inhibited the mRNA expression of ANGPTL8 (**Figures 5D** **, E**).

Because estrogen synthesis is significantly enhanced by a high-fat diet (22), we speculate that the effects of ANGPTL8 in promoting ectopic deposition of lipids induced by HFD in female mice were counteracted by inhibition of its expression by estrogen. We performed OVX on female mice to induce estrogen deficiency (**Figure 5F**). As expected, the qPCR analysis found that the expression of ANGPTL8 increased in the livers of the operation group mice compared to the sham controls at 8 weeks after OVX (**Figure 5G**). OVX significantly increased body weight and ectopic lipid accumulation in WT and ANGPTL8 KO mice. However, body weight and ectopic lipid accumulation were significantly greater in WT mice than in ANGPTL8 KO mice following OVX (**Figures 5H** **–L**). These experiments clearly revealed that estrogen decreases ANGPTL8 expression, and the effects of ANGPTL8 in promoting ectopic lipid accumulation induced by HFD in female mice were counteracted by inhibition of its expression by estrogen.

### ANGPTL8 inhibits the Wnt/β-Catenin signaling pathway and promotes adipogenic differentiation of MSCs

**Figure 6:**
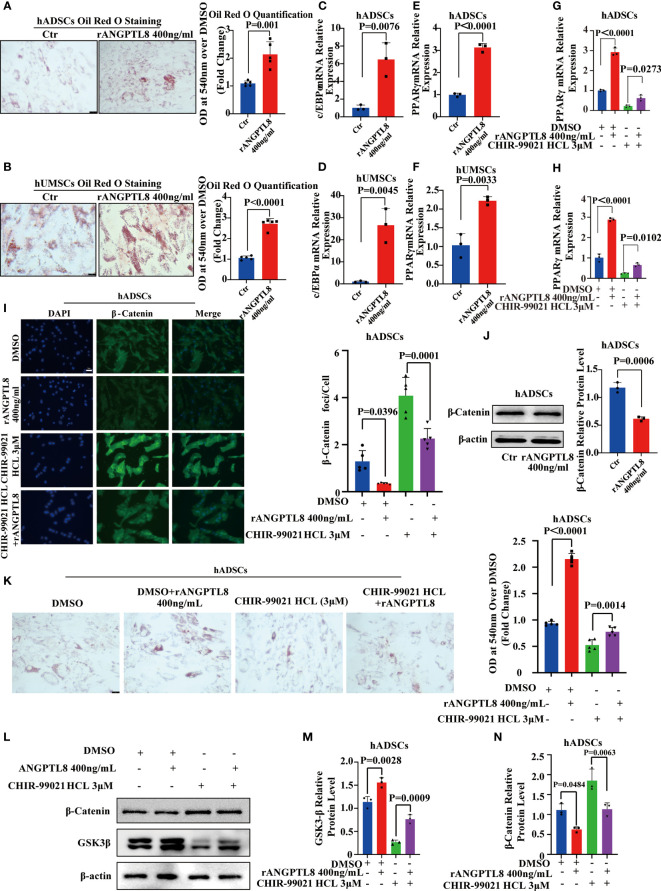
ANGPTL8 inhibits the Wnt/β-catenin signaling pathway and promotes adipogenic differentiation of MSCs. **(A)** Micrographs showing Oil Red O staining of hADSCs after 14 days of differentiation induction and treatment with 400 ng/ml rANGPTL8 (n = 5). **(B)** Micrographs showing Oil Red O staining of hUMSCs after 14 days of differentiation and treatment with 400 ng/ml rANGPTL8 (n = 5). **(C)** Analysis of the relative mRNA expression of the adipogenic gene c/EBPα after 14 days of 400 ng/mL rANGPTL8 treatment during adipogenic differentiation of hADSCs (n = 3). **(D)** Analysis of the relative mRNA expression of the adipogenic gene c/EBPα after 14 days of 400 ng/mL rANGPTL8 treatment during adipogenic differentiation of hUMSCs (n = 3). **(E)** Analysis of the relative mRNA expression of the adipogenic gene PPARγ after 14 days of 400 ng/mL rANGPTL8 treatment during adipogenic differentiation of hADSCs (n = 3). **(F)** Analysis of the relative mRNA expression levels of the adipogenic gene PPARγ after 14 days of 400 ng/mL rANGPTL8 treatment during adipogenic differentiation of hUMSCs (n = 3). **(G)** Analysis of the relative mRNA expression of the adipogenic gene c/EBPα after 14 days of 400 ng/mL rANGPTL8 and 3 μM CHIR-99021 HCl treatment during adipogenic differentiation of hADSCs (n = 3). **(H)** Analysis of the relative mRNA expression of the adipogenic gene PPARγ after 14 days of 400 ng/mL rANGPTL8 and 3 μM CHIR-99021 HCl treatment during adipogenic differentiation of hADSCs (n = 4). **(I)** Representative immunofluorescence staining of β-catenin foci in hADSCs after 14 days of 400 ng/mL rANGPTL8 and 3 μM CHIR-99021 HCl treatment during adipogenic differentiation of hADSCs (n = 5). **(J)** Analysis of the key gene β-catenin in the Wnt/β-catenin signaling pathway after 14 days of 400 ng/mL rANGPTL8 treatment during adipogenic differentiation of hUMSCs by Western blot and densitometric analysis of the separated protein bands (n = 3). **(K)** Shown are Oil Red O-stained hADSCs after 14 days of differentiation induction and treatment with 400 ng/ml rANGPTL8 and 3 μM CHIR-99021 HCl (n = 5). **(L–N)** Analysis of the key gene β-catenin in the Wnt/β-catenin signaling pathway after 14 days of 400 ng/mL rANGPTL8 and 3 μM CHIR-99021 HCl treatment during adipogenic differentiation of hUMSCs by Western blot and densitometric analysis of the separated protein bands (n = 3).

Ectopic lipid accumulation is a metabolic disease characterized by an increase in adipocyte volume and number, in which abnormal adipocyte differentiation leading to increased adipocytes is one of the key contributors to the development of obesity. Studies have shown that MSCs can differentiate into adipogenic, osteogenic, and chondrogenic cells, and their differentiation tropism is closely related to their survival microenvironment. To explore whether ANGPTL8, as a secreted protein that promotes ectopic lipid accumulation, is associated with its regulation of adipogenic differentiation of MSCs, we isolated and characterized hADSCs and hUMSCs from adipose tissue and human umbilical cord (**Supplementary Figures 3A, B**). The expression level of ANGPTL8 mRNA was detected by RT–PCR and hADSCs and hUMSCs were not expressed (**Supplementary Figures 3C, D**). As shown in **Figures 6A–F**, the addition of ANGPTL8 recombinant protein rANGPTL8) to the growth media of hADSCs and hUMSCs significantly enhanced adipogenic differentiation and upregulated the expression of PPARγ and c/EBPα in both cell lines. We also found that ANGPTL8 KO suppressed PPARγ and c/EBPα mRNA expression in the heart, liver, kidney, and white adipose tissue of NCD- and HFD-fed 20 M male mice (**Supplementary Figures 4A–D**).

The Wnt/β-Catenin signaling pathway is a classic pathway regulating the adipogenic differentiation of MSCs (23). Then, we performed western blotting, immunofluorescence, and qPCR to analyze the effect of rANGPTL8 on this signaling pathway. As shown in **Supplementary Figure 4E–I** and **Figures 6G–N**, rANGPTL8 inhibited the expression of the β-catenin protein to promote adipogenic differentiation of hADSCs. rANGPTL8 protein could prevent the activation of LiCl (an activator of the Wnt/β-catenin signaling pathway) and GSK3β inhibitor (CHIR-99021) on the Wnt/β-catenin signaling pathway, decrease GSK3β expression and increase β-catenin protein expression and PPARγ and c/EBPα mRNA expression to restore adipogenic differentiation of hADSCs.

## Discussion

Ectopic fat accumulation leads to the development of a cluster of disorders (24), such as insulin resistance (25), type 2 diabetes, and nonalcoholic fatty liver (26). Improving adipocyte function with thiazolidinediones (TZDs) is a well-established treatment for these disorders (27, 28). However, the side effects of TZDs, such as increased fluid retention and fracture risk, limit their clinical application (29). Therefore, it is important to find new targets for the treatment of ectopic fat deposition to safely and effectively prevent diseases related to obesity, such as type 2 diabetes and nonalcoholic fatty liver disease.

Previous studies have shown that ANGPTL8, which is expressed in liver and adipose tissue, is required to redirect dietary TG from oxidative to storage tissues following food intake (30). ANGPTL8 general knockout suppresses plasma triglyceride concentrations and adiposity in mice and rats (31). Plasma ANGPTL8 concentrations are associated with obesity (32), type 2 diabetes (33), and nonalcoholic fatty liver (34). We analyzed the transcriptome data (21) of subcutaneous adipose tissue of obese patients and found that the expression of ANGPTL8 was significantly higher than the expression of ANGPTL8 of the control (healthy group), and consistent results were obtained in the liver of a high-fat diet (HFD)-induced mouse obesity model. Therefore, we speculate that ANGPTL8 may play an important role in ectopic fat metabolism.

Studies have found that ANGPTL8 knockout suppresses short-term HFD-induced weight gain, mainly because ANGPTL8 knockout reduces adipose tissue weight gain rather than ectopic fat accumulation in other organs (35). However, the accumulation of ectopic fat is a long-term process, so the effect of ANGPTL8 on long-term diet-induced obesity, especially on lipid deposition in various organs, remains unclear. We found that ANGPTL8 KO induced body weight and organ weight differences through 20-month NCD and HFD diet induction, which was caused by reduced lipid deposition in the heart, liver, kidney, and WAT of male mice. In addition, our results showed that Oil Red O combined with Sudan Black B staining could better identify the distribution of lipids than single dye staining. Although Oil Red O staining is a classical method to identify lipid deposits (36), it has a poor recognition ability for diffusely distributed lipids, such as scattered neutral fat and phospholipids, and Sudan black B has a better recognition effect for diffusely distributed lipids in cells (37). Combining these two methods, we found that ANGPTL8 could affect lipid deposition in more organs.

In addition, our results indicate that ANGPTL8 KO female mice, especially the HFD-fed group, had no differences in body weight or ectopic lipid deposition. Many men are more obese than women due to the protective effects of estrogen (30), but the underlying mechanism needs further study. Our results showed that the protective effect of estrogen on body obesity is related to ANGPTL8. Female ANGPTL8 KO mice on a normal diet were significantly lighter than WT mice, and the difference was manifested mainly in the difference in liver and WAT weights. HFD can significantly promote estrogen synthesis (22), and our results showed that there was no significant difference in body weight or visceral weight between ANGPTL8 KO female and WT mice. Oldoni et al. showed that ANGPTL8 from the liver and adipose tissue has different roles in the process of lipid metabolism; they found that mice lacking hepatic ANGPTL8 have no circulating ANGPTL8, high intravascular LPL activity, low plasma TG levels, and evidence of decreased delivery of dietary lipids to adipose tissue. In contrast, mice lacking ANGPTL8 in adipose tissue have higher postprandial TG levels and similar intravascular LPL activity and plasma ANGPTL8 levels, and higher levels of plasma TG. Their results suggest that hepatic secretion of ANGPTL8 is the main source of its role in regulating lipid metabolism and obesity (38). Therefore, we examined the regulation of ANGPTL8 expression in hepatocytes by estrogen, and our *in vitro* and *in vivo* experiments confirmed that estrogen could inhibit the expression of ANGPTL8 in hepatocytes. These results suggest that HFD induces WT female mice to secrete more estrogen to suppress ANGPTL8 expression and counteract the effects of ANGPTL8 knockout on obesity and lipid deposition.

Increased adipocyte number is an important contributor to weight differences and ectopic fat deposition. Studies have shown that ANGPTL8 promotes adipocyte differentiation (14), but some studies interfere with ANGPTL8 expression in adipocytes, and there is no difference in adipocyte number (39). Therefore, it is unclear whether ANGPTL8 induces obesity by affecting adipocyte number.

Adipocytes are differentiated from MSCs mainly in tissues (40), and the abnormal enhancement of adipogenic differentiation under the induction of obesogens will cause obesity (10). We found that the rANGPTL8 protein could promote the differentiation of hADSCs and human hUMSCs into adipocytes by upregulating the key genes PPARγ and c/EBPα of adipogenic differentiation. These findings suggest that ANGPTL8 is a novel obesogen.

The Wnt/β-catenin signaling pathway is a classic pathway regulating the adipogenic differentiation of MSCs (23). Our data support that the rANGPTL8 protein inhibits the expression of the β-catenin protein to promote adipogenic differentiation of ADMSCs. LiCl inhibits adipogenic differentiation of MSCs by activating the Wnt/β-catenin signaling pathway (41). rANGPTL8 protein could prevent the activation of LiCl on the Wnt/β-catenin signaling pathway, decrease β-catenin protein expression and upregulate PPARγ and c/EBPα mRNA expression to restore adipogenic differentiation of hADSCs. We provided several novel findings demonstrating that targeted inhibition of ANGPTL8 may be beneficial in alleviating ectopic lipid deposition. Estrogen can significantly inhibit the expression of ANGPTL8 in the liver, which explains why men are more prone to obesity than women and suggests that some diseases in men caused by abnormal fat metabolism may be prevented by blocking the expression of ANGPTL8.

**Figure 7:**
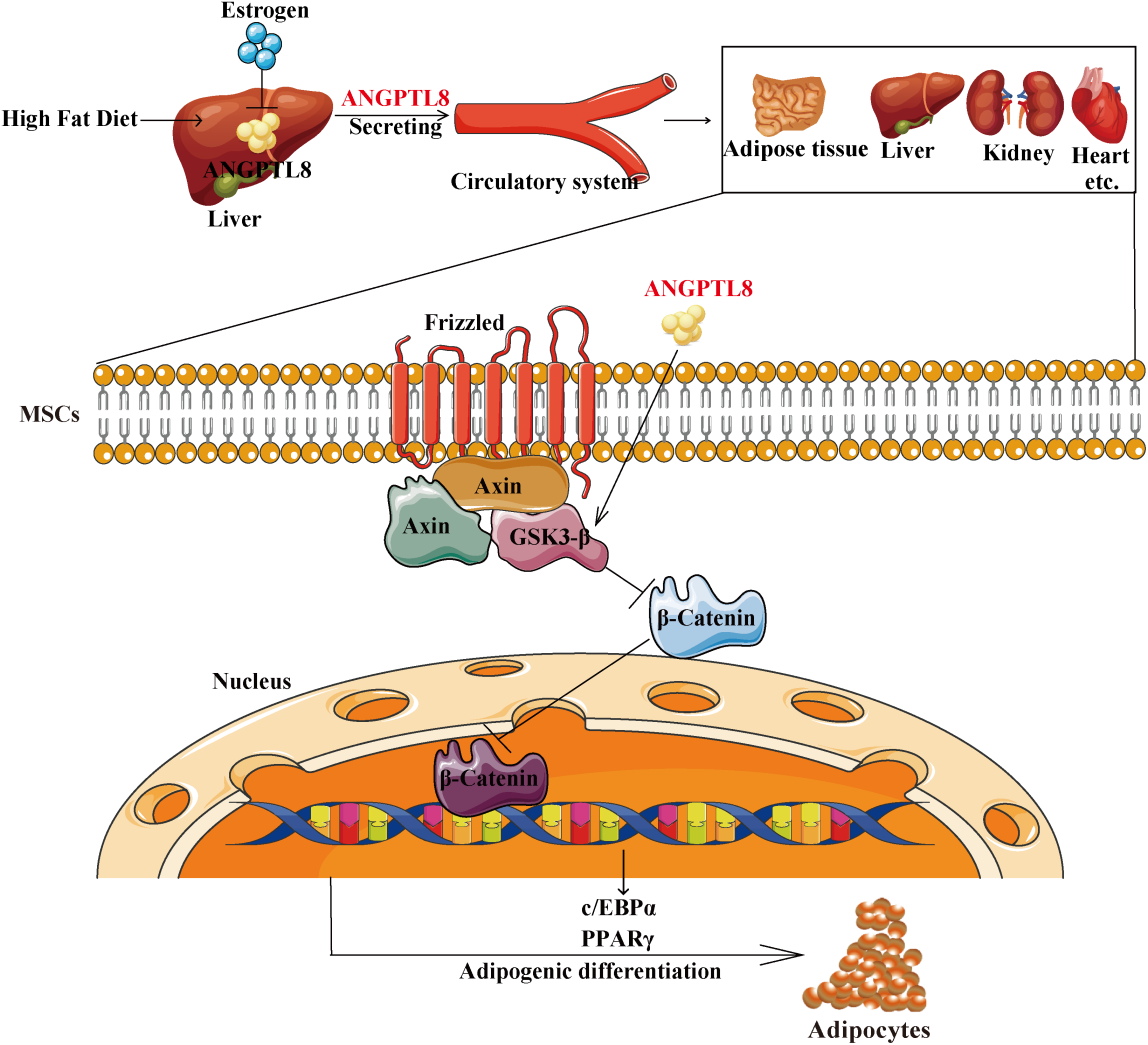
Schematic representation of the proposed mechanism by which ANGPTL8 induces ectopic lipid deposition by promoting adipogenic differentiation of mesenchymal stem cells. The elevated fatty acid levels upon long-term exposure to an HFD upregulate liver ANGPTL8. ANGPTL8, in turn, secreted by the liver into the blood, acts on MSC cells in various organs. Secreted ANGPTL8 upregulates the expression of PPARγ and c/EBPα by inhibiting the Wnt/β-catenin signaling pathway and promotes the adipogenic differentiation of MSCs, resulting in lipid deposition in multiple organs and causing obesity in male mice. Estrogen can inhibit the expression of ANGPTL8 to counteract the effect of ANPTL8 KO on obesity in female mice.

Our findings ultimately support a model in which ANGPTL8 upregulated the expression of PPARγ and c/EBPα by inhibiting the Wnt/β-catenin signaling pathway and promoting the adipogenic differentiation of MSCs, resulting in lipid deposition in multiple organs and causing obesity in male mice. Estrogen can inhibit the expression of ANGPTL8 to counteract the effect of ANPTL8 KO on obesity in female mice (**Figure 7**). In conclusion, our study indicates that ANGPTL8 may be a new target for the prevention and treatment of ectopic lipid deposition in males.

## Data availability statement

The datasets presented in this study can be found in online repositories. The names of the repository/repositories and accession number(s) can be found below: https://www.ncbi.nlm.nih.gov/geo/, GSE156906; https://www.ncbi.nlm.nih.gov/geo/, GSE55668.

## Ethics statement

The studies involving human participants were reviewed and approved by Ethics Committee of Taihe Hospital, China. The patients/participants provided their written informed consent to participate in this study. The animal study was reviewed and approved by Ethics committee of Hubei University of Medicine, China.

## Author contributions

JT and SM contributed to data curation, investigation, and methodology. YY and YF contributed to writing-review and editing. JT, FZ, YG, LH, CG, LY, YC, and QZ contributed to verifying all the experimental results. XG contributed to writing the original draft and funding acquisition. All authors approved the final version of the manuscript.

## Funding

This research was funded by the National Natural Science Foundation of China (No. 82073232, 82101632, 81700769, 81641028), the Hubei Science & Technology Department Foundation (2020CFB558, 2018ACA162), the Key Projects of Hubei Education (D20202103), the Department of Biomedical Research Foundation, Hubei University of Medicine (HBMUPI201803), the Innovative Research Program for Graduates of Hubei University of Medicine (YC2021002, YC2020039, YC2020002, YC2019003, YC2019008), and the Advantages Discipline Group (medicine) project in Higher Education of Hubei Province (2022XKQT3, 2022XKQY1).Scientific research project of Shiyan science and Technology Bureau (21Y06, 21Y38).

## Conflict of interest

The authors declare that the research was conducted in the absence of any commercial or financial relationships that could be construed as a potential conflict of interest.

## Publisher’s note

All claims expressed in this article are solely those of the authors and do not necessarily represent those of their affiliated organizations, or those of the publisher, the editors and the reviewers. Any product that may be evaluated in this article, or claim that may be made by its manufacturer, is not guaranteed or endorsed by the publisher.
