# Corrigendum: ANGPTL8 promotes adipogenic differentiation of mesenchymal stem cells: potential role in ectopic lipid deposition

**DOI:** 10.3389/fendo.2025.1617549

**Published:** 2025-05-28

**Authors:** Jian Tang, Shinan Ma, Yujiu Gao, Fan Zeng, Ying Feng, Chong Guo, Lin Hu, Lingling Yang, Yanghui Chen, Qiufang Zhang, Yahong Yuan, Xingrong Guo

**Affiliations:** ^1^ Department of Neurosurgery, Hubei Key Laboratory of Embryonic Stem Cell Research, Taihe Hospital, Hubei University of Medicine, Shiyan, China; ^2^ Central Laboratory, Xiangyang Central Hospital, Affiliated Hospital of Hubei University of Arts and Science, Xiangyang, China; ^3^ Department of Geriatrics & General Medicine, Affiliated Taihe Hospital of Hubei University of Medicine, Shiyan, China; ^4^ Hubei Clinical Research Center for Umbilical Cord Blood Hematopoietic Stem Cells, Taihe Hospital, Hubei University of Medicine, Shiyan, Hubei, China

**Keywords:** ectopic lipid accumulation, Wnt/β-Catenin pathway, ANGPTL8, mesenchymal stem cells, adipogenic differentiation

In the published article, there was an error in the legend of **Figure 4** as published. “(L) Representative HE staining of white adipose tissue of female WT and ANGPTL8 KO mice after HFD consumption for 20 months.” is redundant. The corrected legend appears below.

In the published article, there were three errors in **Figure 4** as published. In **Figure 4C**, the annotation “HFD20MFemaleMice” was incorrect; In **Figure 4H**, the representative images of the Sudan Black B staining for the liver tissue in the WT group were uploaded incorrectly; In **Figure 4K**, the annotation “HFD 20M Mice Sudan Black B Staining” was incorrect. The corrected **Figure 4** appear below.

**Figure 4:**
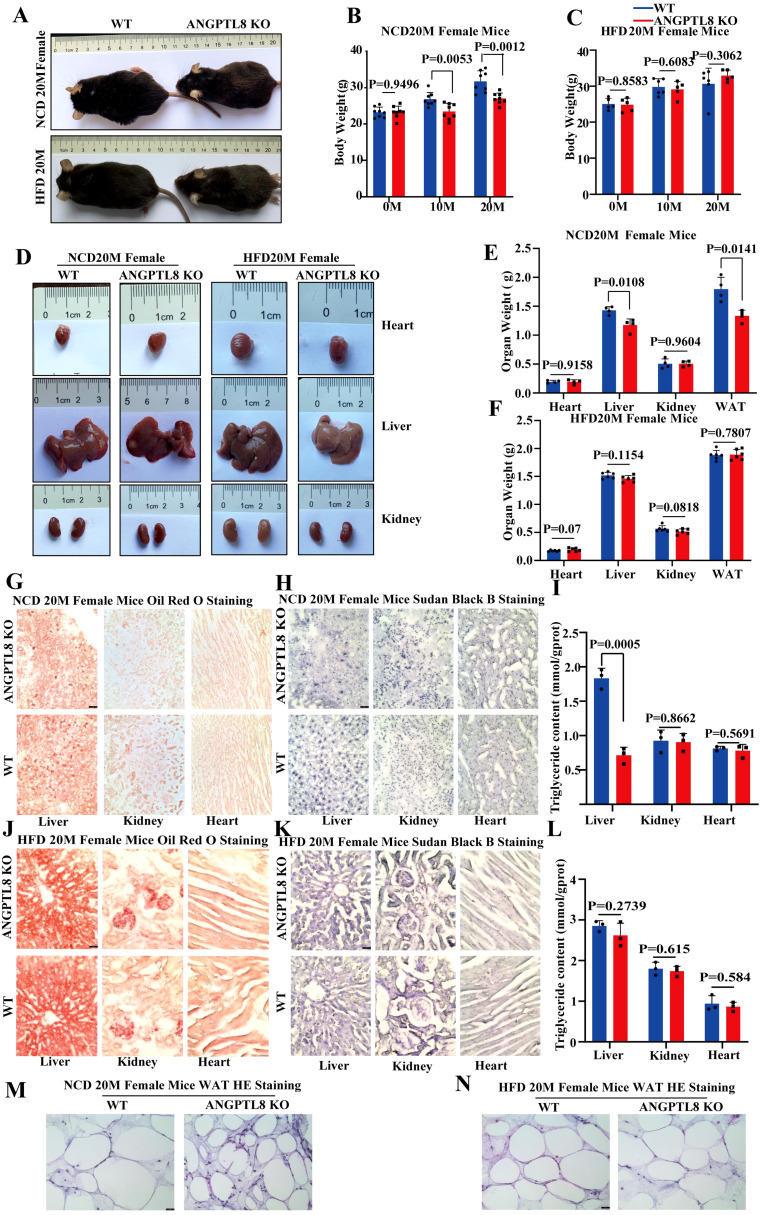
The effect of ANGPTL8 on diet-induced obesity and lipid deposition in female mice. **(A)** Gross appearance of ANGPTL8 KO and WT female mice fed an NCD or HFD for 20 months. **(B, C)** Body weight change of WT and ANGPTL8 KO female mice at 10 and 20 months after NCD **(B)** (n = 8) and HFD feeding (n ≥ 5). **(D)** Macroscopic heart, liver, and kidney appearance of ANGPTL8 KO and WT female mice fed an NCD or HFD for 20 months. **(E, F)** The weights of the heart, liver, kidney, and white adipose tissue of ANGPTL8 KO and WT female mice fed an NCD (n = 4) and HFD for 20 months (n = 6). **(G)** Micrographs showing Oil Red O staining of the frozen liver, kidney, and heart sections in female WT and ANGPTL8 KO mice after NCD feeding for 20 months (red = Oil Red O). **(H)** Micrographs showing Sudan Black B staining of the frozen liver, kidney, and heart sections from female WT and ANGPTL8 KO mice after NCD feeding for 20 months. **(I)** Quantitative analysis of TG in the liver, kidney, and heart of ANGPTL8-KO and WT female mice treated with NCD for 20 months. **(J)** Micrographs showing Oil Red O staining of frozen liver, kidney, and heart sections from female WT and ANGPTL8 KO mice after HFD consumption for 20 months. **(K)** Micrographs showing Sudan Black B staining on frozen liver, kidney, and heart sections in female WT and ANGPTL8 KO mice after HFD consumption for 20 months. **(L)** Quantitative analysis of TG in the liver, kidney, and heart of ANGPTL8-KO and WT female mice fed an HFD for 20 months. **(M, N)** Representative HE staining of white adipose tissue of female WT and ANGPTL8 KO mice after NCD and HFD feeding for 20 months. (red = Oil Red O. (scale bar = 50 μm, n = 5).

The authors apologize for these error and state that this does not change the scientific conclusions of the article in any way. The original article has been updated.

