# Supplementary figures and images for: ANGPTL8 promotes adipogenic differentiation of mesenchymal stem cells: potential role in ectopic lipid deposition
Source: Front Endocrinol (Lausanne). 2022 Aug 11;13:927763. doi: 10.3389/fendo.2022.927763 (PMC9404696; doi:10.3389/fendo.2022.927763)

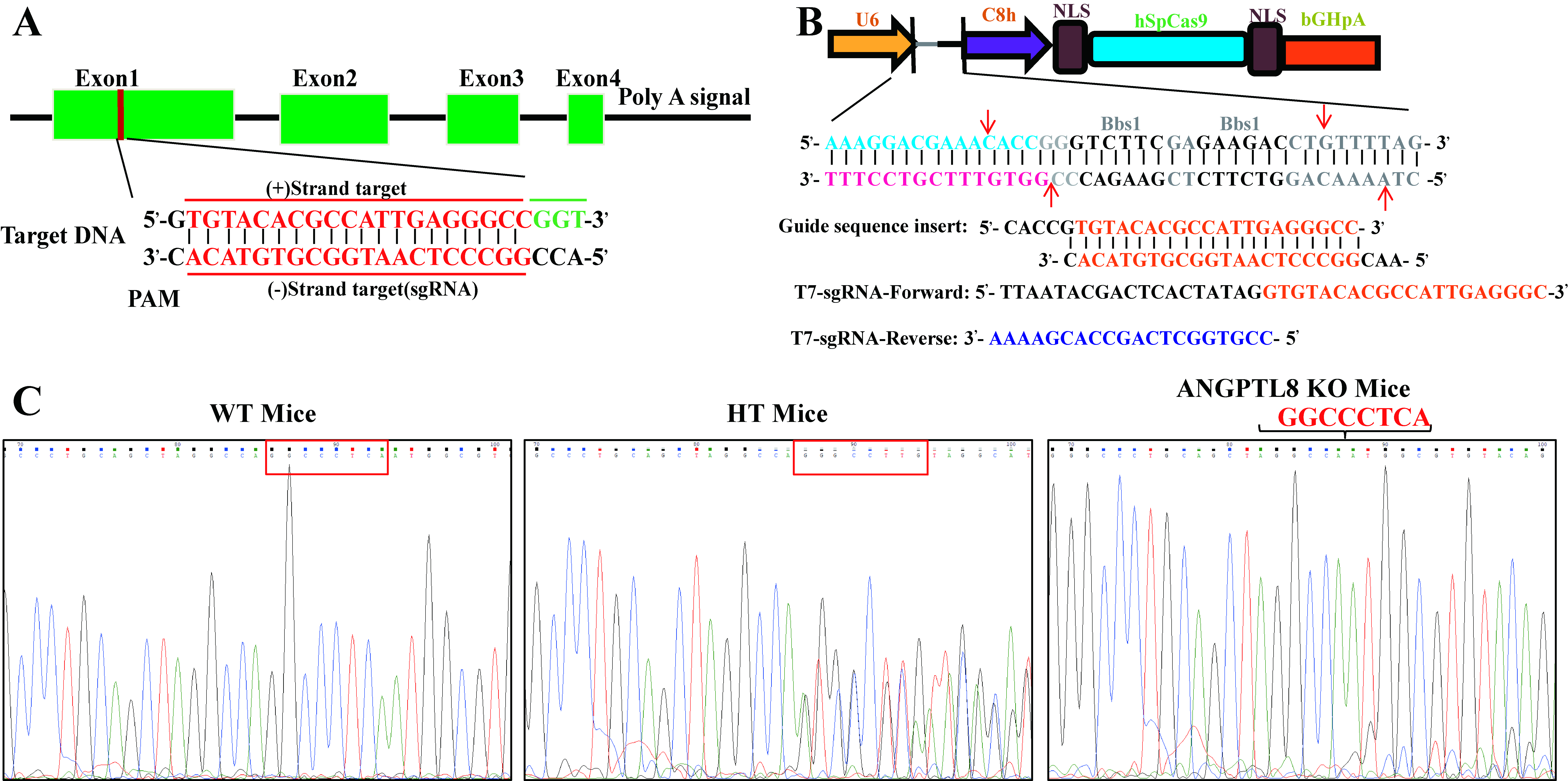

Supplement: Supplementary file 1 [file Image_1.tif]

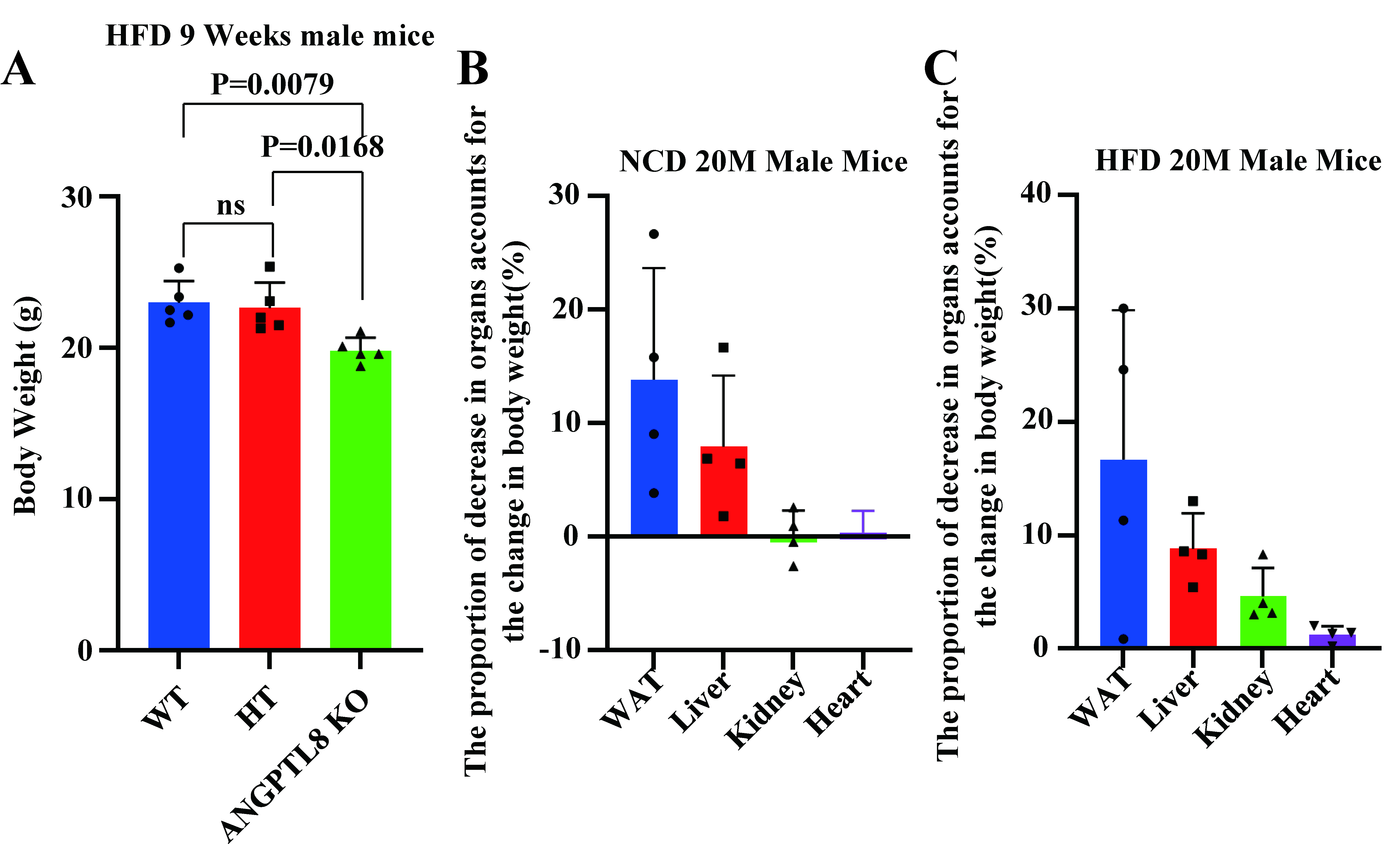

Supplement: Supplementary file 2 [file Image_2.tif]

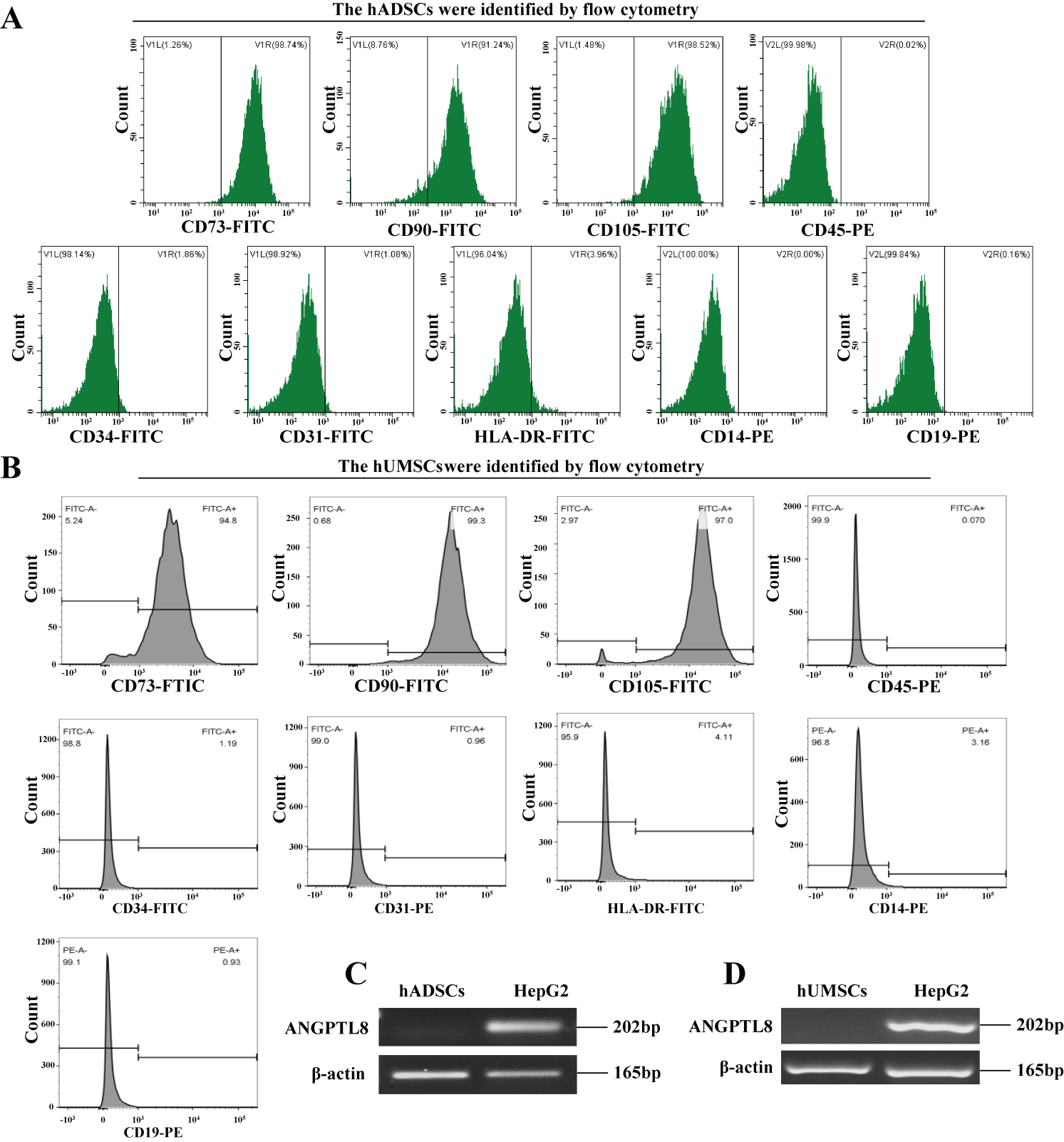

Supplement: Supplementary file 3 [file Image_3.tif]

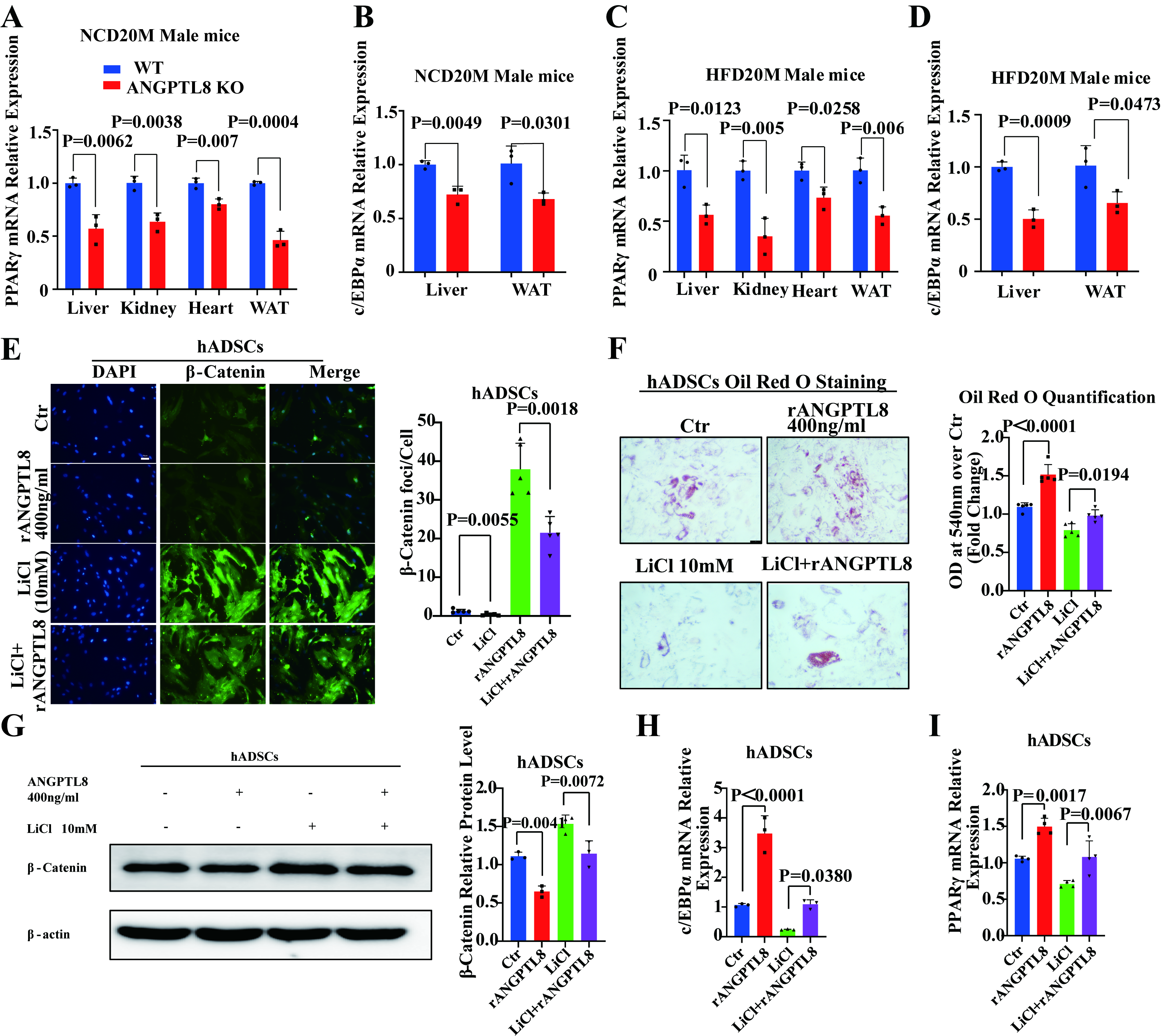

Supplement: Supplementary file 4 [file Image_4.tif]
