# Supplementary material for: ANGPTL8 promotes adipogenic differentiation of mesenchymal stem cells: potential role in ectopic lipid deposition
Source: Front Endocrinol (Lausanne). 2022 Aug 11;13:927763. doi: 10.3389/fendo.2022.927763 (PMC9404696; doi:10.3389/fendo.2022.927763)
Supplement: Supplementary file 5 [file Table_1.docx]

Supplementary Material

# Supplementary Figure 1

# Supplementary Figure 1. ANGPTL8−/− mice were generated using the CRISPR/Cas9 30 system. (A) The designed sgRNA consisted of the DNA target sequence (red letters) directly 2 upstream of a 5′-TGG adjacent motif (PAM, green letters). (B) Schematic diagram showing the guide sequence inserted into the pX330 plasmid (between the red arrows). The guide sequence included overhangs (black letters: 5′-CACC-3′, 5-AAC-3′) for ligation into the BbSI sites (bold and black letters) in pX330. The T7 promoter was added to primers for PCR amplification using the pX330-Cas9-ANGPTL8-1-sgRNA constructs as the template. The PCR products were then transcribed into sgRNA containing ANGPTL8 sgRNA (red letters) and tracer RNA (purple letters). (C) Sequencing analysis of base deletion in ANGPTL8 knockout (KO) homozygous mice (the red marker is the missing base).

# Supplementary Figure 2

**Supplementary Figure 2** Effects of ANGPTL8 on the body weight and organ weight of male mice. **(A)** Analysis of body weight change of WT, HT and ANGPTL8 KO male mice after NCD feeding for 9 weeks (n = 5)**.** (B) Analysis of organ (WAT, liver, kidney and heart) weight loss as a proportion of body weight loss in NCD20 M mice. (C) Analysis of organ (WAT, liver, kidney and heart) weight loss as a proportion of body weight loss in HFD20 M mice.

# Supplementary Figure 3

**Supplementary Figure 3.** Isolation and identification of mesenchymal stem cells**.** (A) and (B) Flow cytometry analyses of mesenchymal hADSCs and hUMSCs for minimal criteria cell surface marker expression (CD73+, CD90+, CD105+, CD45-, CD34, CD31-, HLA‐DR-, CD14-, CD19-). (C) Detection of ANGPTL8 gene expression in hADSCs by RT–PCR. (D) Detection of ANGPTL8 gene expression in hUMSCs by RT–PCR.

# Supplementary Figure 4

**Supplementary Figure 4.** Mechanism of ANGPTL8 promoting adipogenic differentiation of mesenchymal stem cells. (A) & (B) Analysis of the effect of ANGPTL8 on the expression of PPARγ and c/EBPα in the liver, kidney, heart and WAT of NCD20 M mice by RT–qPCR. (C) & (D) Analysis of the effect of ANGPTL8 on the expression of PPARγ and c/EBPα in the liver, kidney, heart and WAT of HFD20 M mice by RT–qPCR. (E) & (F) Representative immunofluorescence staining of β-catenin foci in hADSCs after 14 days of 400 ng/mL rANGPTL8 and 10 mM LiCl treatment during adipogenic differentiation of hADSCs (n=5). (G) & (H) Oil Red O-stained hADSCs after 14 days of differentiation induction and treatment with 400 ng/ml rANGPTL8 and 10 mM LiCl (n=5). (I) & (J) Analysis of the relative mRNA expression of the adipogenic genes PPARγ and c/EBPα after 14 days of 400 ng/mL rANGPTL8 and 10 mM LiCl treatment during adipogenic differentiation of hADSCs (n = 3). (K) & (L) Analysis of the key gene β-catenin in the Wnt/β-catenin signaling pathway after 14 days of 400 ng/mL rANGPTL8 and 10 mM LiCl treatment during adipogenic differentiation of hUMSCs by Western blot and densitometric analysis of the separated protein bands (n=3).
